# Supplementary material for: Parallel wave-based analog computing using metagratings
Source: Nanophotonics. 2022 Mar 24;11(8):1561–71. doi: 10.1515/nanoph-2021-0710 (PMC9125804; doi:10.1515/nanoph-2021-0710)
Supplement: Supplementary file 1 — Supplementary Material Details [file j_nanoph-2021-0710_suppl.pdf]

Supplementary Information for

Parallel wave-based analog computing using metagratings

Hamid Rajabalipanah[1], Ali Momeni[2], Mahdi Rahmanzadeh[1], Ali Abdolali [1]*, Romain Fleury[2]*.

[1] Applied Electromagnetic Laboratory, School of Electrical Engineering, Iran University of Science and Technology, Tehran, 1684613114, Iran

[2] Laboratory of Wave Engineering, School of Electrical Engineering, Swiss Federal Institute of Technology in Lausanne (EPFL), Lausanne, Switzerland.

The Supplementary file includes:

The detailed explanations of A) Theoretical Analysis, B) Allowed Space Harmonics, C) Parallel Signal Processing, D) Sensitivity Analysis, and E) Image Denoising

Supplementary Appendix A (Theoretical Analysis)

Each groove can be modeled by a parallel plate waveguide supporting a single transverse electromagnetic (TEM) wave below the cutoff frequency $f_c = c/2\max[w_1 n_1, w_2 n_2]$. Subsequently, we derive the tangential electric fields according to Maxwell's equations as a superposition of forward and backward propagating plane waves inside each of grooves and use mode matching on the aperture z=0 to retrieve the scattering coefficients and vanishing of the tangential electric field at the PEC interface:

$$H^{y}_{\text{grv1}} = \sum H^{\pm}_{0,\text{grv1}} e^{\pm j\beta_{\text{grv1}} z} \tag{S1}$$

$$E^{x}_{\text{grv1}} = \sum (-1)^{\pm} Y_{0,\text{grv1}} H^{\pm}_{0,\text{grv1}} e^{\pm j\beta_{\text{grv1}} z} \tag{S2}$$

for $x \in \text{grv1}$, and

$$H^{y}_{\text{grv2}} = \sum H^{\pm}_{0,\text{grv2}} e^{\pm j\beta_{\text{grv2}} z} \tag{S3}$$

$$E^{x}_{\text{grv2}} = \sum (-1)^{\pm} Y_{0,\text{grv2}} H^{\pm}_{0,\text{grv2}} e^{\pm j\beta_{\text{grv2}} z} \tag{S4}$$

for $x \in \text{grv2}$, where $\beta_{\text{grvi}} = k_0 n_i \sqrt{1+(1-j)\delta_s / w_i}$ and $Y_{0,\text{grv,i}} = \beta_{\text{grvi}} / \omega\varepsilon_0 n_i^2$ $(i=1,2)$. are the propagation constant and the admittance of the TEM mode involved inside the first and second grooves. The propagation constant incorporates the ohmic losses of the lossy parallel plate waveguide by making a good conductor approximation with a strong skin effect condition $\delta_s = \sqrt{2/\omega\mu_0\sigma}$. The continuity of total tangential electric field on the whole surface of each period yields:

$$1-\tilde{R}_0 = \frac{Y_{0,\text{grv2}}}{Y_0} H^{+}_{0,\text{grv2}} M^{0}_{\text{grv2+}} - \frac{Y_{0,\text{grv2}}}{Y_0} H^{-}_{0,\text{grv2}} M^{0}_{\text{grv2+}} + \frac{Y_{0,\text{grv1}}}{Y_0} H^{+}_{0,\text{grv1}} M^{0}_{\text{grv1+}} - \frac{Y_{0,\text{grv1}}}{Y_0} H^{-}_{0,\text{grv1}} M^{0}_{\text{grv1+}} \tag{S5}$$

$$\tilde{R}_{m\neq 0} = -\frac{Y_{0,\text{grv1}}}{Y_m} H^{+}_{0,\text{grv1}} M^{m}_{\text{grv1+}} + \frac{Y_{0,\text{grv1}}}{Y_m} H^{-}_{0,\text{grv1}} M^{m}_{\text{grv1+}} - \frac{Y_{0,\text{grv2}}}{Y_m} H^{+}_{0,\text{grv2}} M^{m}_{\text{grv2+}} + \frac{Y_{0,\text{grv2}}}{Y_m} H^{-}_{0,\text{grv2}} M^{m}_{\text{grv2+}} \tag{S6}$$

where,

$$M_{\text{grv1}\pm}^{m}=\frac{1}{L_x}\int_{\text{grv1}} e^{\pm jk_{xm}x}dx \tag{S7}$$

$$M_{\text{grv2}\pm}^{m}=\frac{1}{L_x}\int_{\text{grv2}} e^{\pm jk_{xm}x}dx \tag{S8}$$

Here, we multiply the electric fields by $e^{jk_{xm}x}$ and take the integral of both sides over one period. Using the boundary conditions of the tangential magnetic field and then, taking the integral of both sides over each slit width, we have:

$$L_x M_{\text{grv1}-}^{0}+L_x\sum_m \tilde{R}_m M_{\text{grv1}-}^{m}=w_1 H_{0,\text{grv1}}^{+}+w_1 H_{0,\text{grv1}}^{-} \tag{S9}$$

$$L_x M_{\text{grv2}-}^{0}+L_x\sum_m \tilde{R}_m M_{\text{grv2}-}^{m}=w_2 H_{0,\text{grv2}}^{+}+w_2 H_{0,\text{grv2}}^{-} \tag{S10}$$

The PEC termination at the end of each groove commands:

$$H_{0,\text{grv1}}^{-}=H_{0,\text{grv1}}^{+}e^{-2j\beta_{\text{grv1}}h_1} \tag{S11}$$

$$H_{0,\text{grv2}}^{-}=H_{0,\text{grv2}}^{+}e^{-2j\beta_{\text{grv2}}h_2} \tag{S12}$$

The detailed expressions for A and B coefficients of Eqs. (4a), (4b) can then be written as:

$$A_m=-M_{\text{grv1}-}^{0}M_{2+}^{m}S_2C_3Y_{0,\text{grv1}}-M_{\text{grv1}-}^{0}M_{2+}^{m}S_2S_3Y_{0,\text{grv1}}Y_{0,\text{grv2}}\sum_q\frac{M_{3+}^{q}M_{3-}^{q}}{Y_{1q}} \tag{S13}$$

$$-M_{3-}^{0}M_{3+}^{m}S_3C_2Y_{0,\text{grv2}}-M_{3-}^{0}M_{3+}^{m}S_2S_3Y_{0,\text{grv1}}Y_{0,\text{grv2}}\sum_q\frac{M_{2+}^{q}M_{\text{grv1}-}^{q}}{Y_{1q}}$$

$$+M_{3-}^{0}M_{2+}^{m}S_2S_3Y_{0,\text{grv1}}Y_{0,\text{grv2}}\sum_q\frac{M_{\text{grv1}-}^{q}M_{3+}^{q}}{Y_{1q}}+M_{\text{grv1}-}^{0}M_{3+}^{m}S_2S_3Y_{0,\text{grv1}}Y_{0,\text{grv2}}\sum_q\frac{M_{2+}^{q}M_{3-}^{q}}{Y_{1q}}$$

$$B = S_2 C_3 Y_{0,\mathrm{grv1}} \sum_q \frac{M_{2+}^q M_{\mathrm{grv1}-}^q}{Y_{1q}} + S_3 C_2 Y_{0,\mathrm{grv2}} \sum_q \frac{M_{3+}^q M_{3-}^q}{Y_{1q}} + C_2 C_3 + \tag{S14}$$

$$S_2 S_3 Y_{0,\mathrm{grv1}} Y_{0,\mathrm{grv2}} \left(\sum_q \frac{M_{2+}^q M_{\mathrm{grv1}-}^q}{Y_{1q}} \sum_q \frac{M_{3+}^q M_{3-}^q}{Y_{1q}} - \sum_q \frac{M_{2+}^q M_{3-}^q}{Y_{1q}} \sum_q \frac{M_{2-}^q M_{3+}^q}{Y_{1q}} \right)$$

$$S_i = (1 - e^{-2j\beta_i h_i}) \quad (i = 2, 3)$$

$$C_i = \frac{w_i}{L_x}(1 + e^{-2j\beta_i h_i}) \quad (i = 2, 3)$$

Supplementary Appendix B (Allowed Space Harmonics)

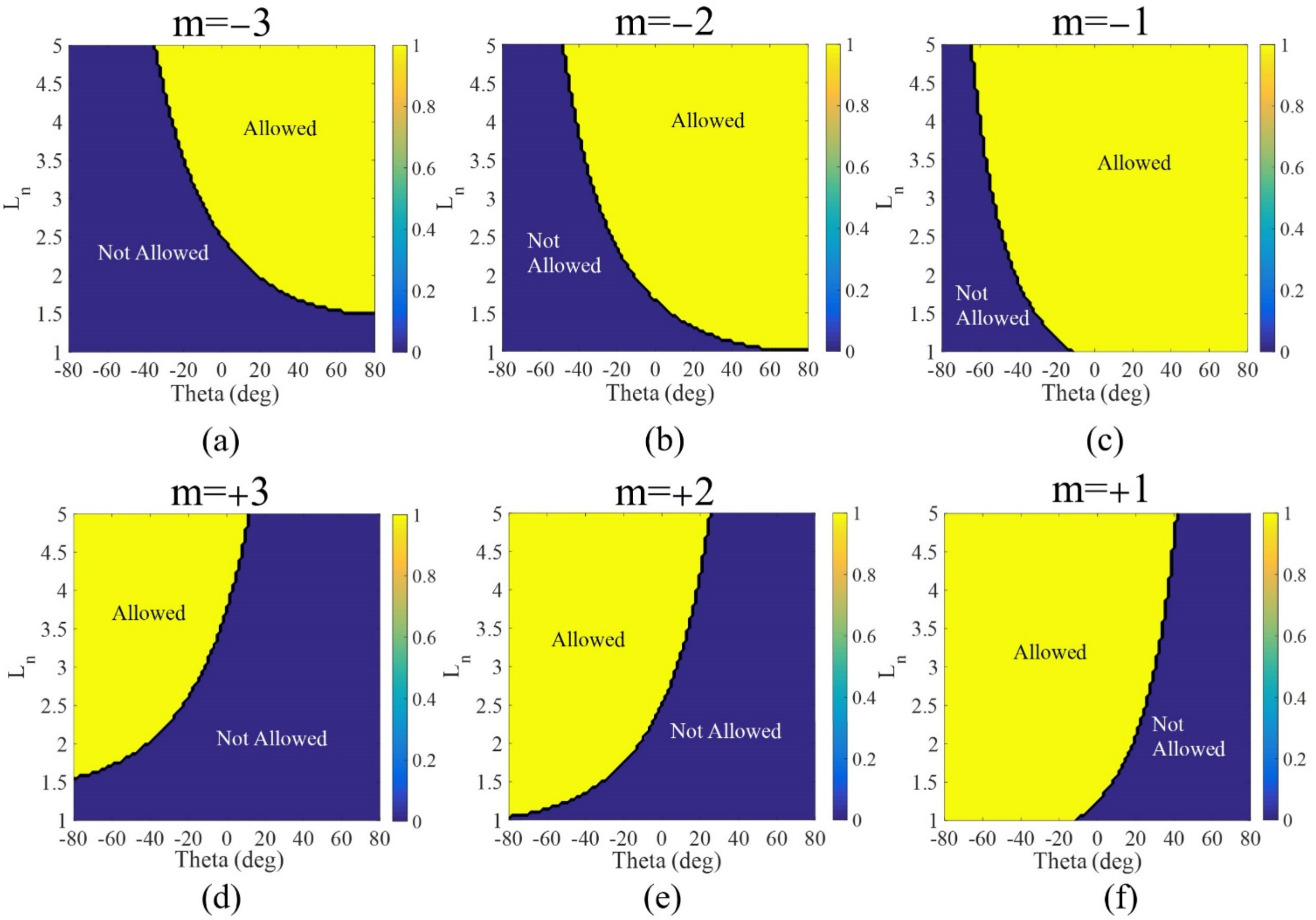

Supplementary Figure S1. The allowed and not allowed processing channels for different incident wave angles and harmonic numbers where the angular beamwidth of the input beam is specified with ζ =0.2.

Supplementary Appendix C (Parallel Signal Processing)

To show the capability of these metagratings, we have designed another structure enabling parallel analog computing without cross-talk between the existing channels. The schematic of illustration is given in **Supplementary Figure S2a** in which the input signals illuminate the structure from ports 2 and 6 while the output signals are reflected through ports 5 and 7, respectively. The realized transfer functions are shown in **Supplementary Figures S2b, c** and the optimized parameters are given in the caption of the same figure. As can be seen, the linear trend of the amplitude curve as well as 180° phase jump are successfully achieved in the vicinity of the channel angles (i.e., 41° and 67°), enabling two different channels for the first-order spatial differentiation at the same time without any cross-talk.

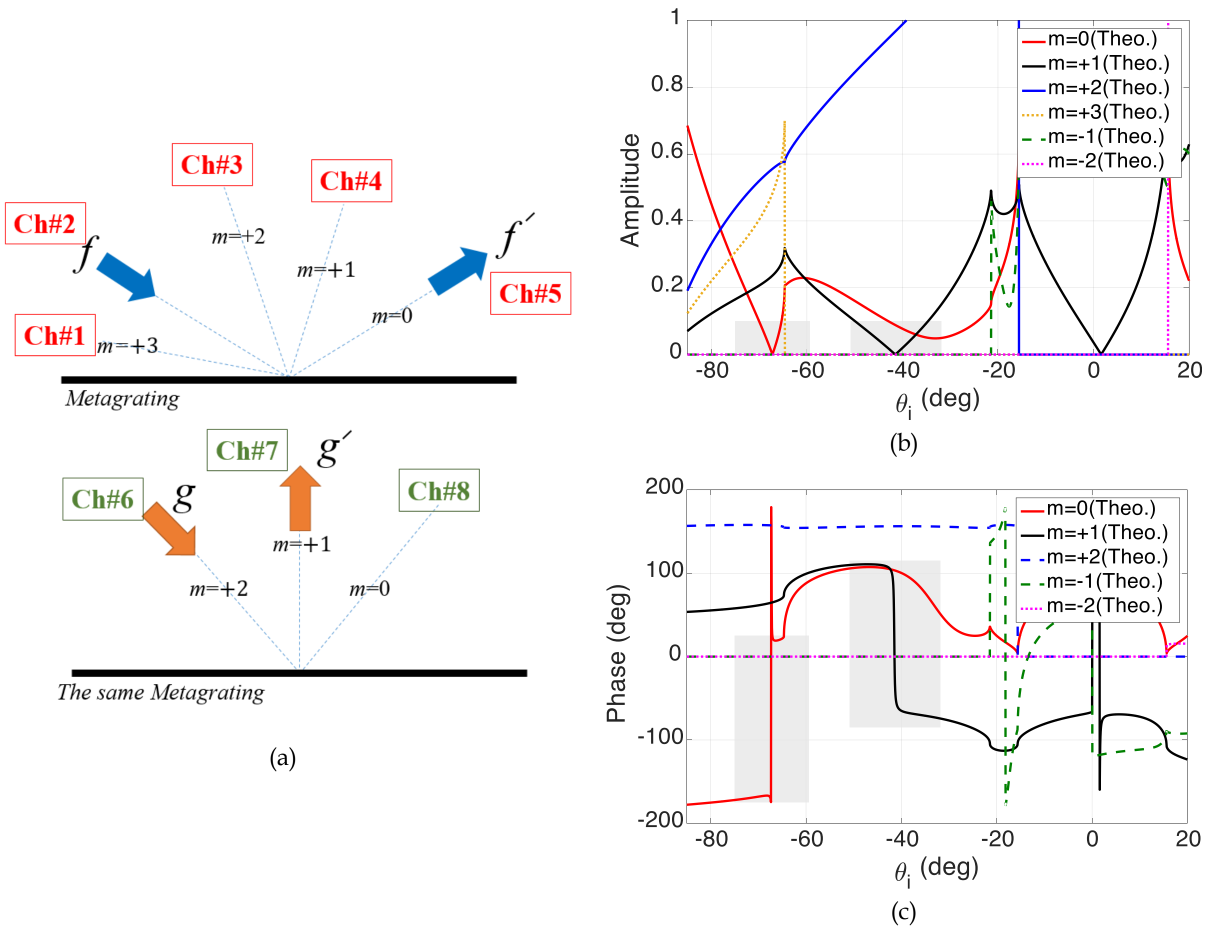

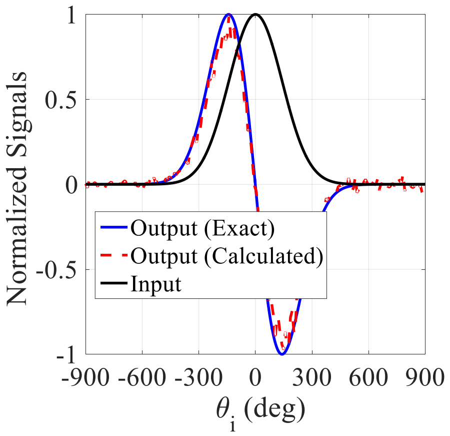

(d)

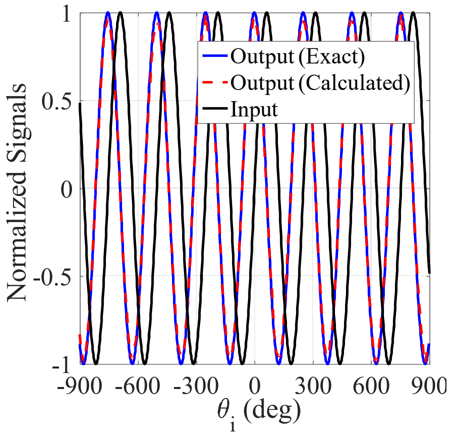

(e)

Supplementary Figure S2. Parallel Signal Processing a) The schematic illustration of a metagrating designed for realizing two separate first-order spatial differentiation channels at the same time. b) The amplitude and c) phase of the space harmonics. (d), (e) The input and output signals of the channels oriented along 41° and 67° directions, respectively. The optimum geometrical parameters are as:Lx=1.576λ(λ is the operating wavelength), w_1=0.042Lx, w_2=0.084Lx, h_1=0.45Lx, h_2=0.75Lx, d=0.4Lx, and ε_{r0}= ε_{r1}= ε_{r2}=1.

Supplementary Appendix D (Sensitivity Analysis)

Here, we present the results of a sensitivity analysis on the metagrating designed in Figure 3. The goal is to show that the proposed metagrating can tolerate small fabrication perturbations. The amplitude and phase of the realized transfer function, i.e., the angular spectra, are computed for different changes in the geometrical parameters. The results illustrated in **Supplementary Figures S3-6**, indicating that small variations in the parameters d, h_1, h_2, w_1, w_2 result in a relatively negligible change in the amplitude and phase of the reflection coefficients pertaining to the zeroth (**Supplementary Figures S3, 4**) and first modes (**Supplementary Figures S5, 6**).

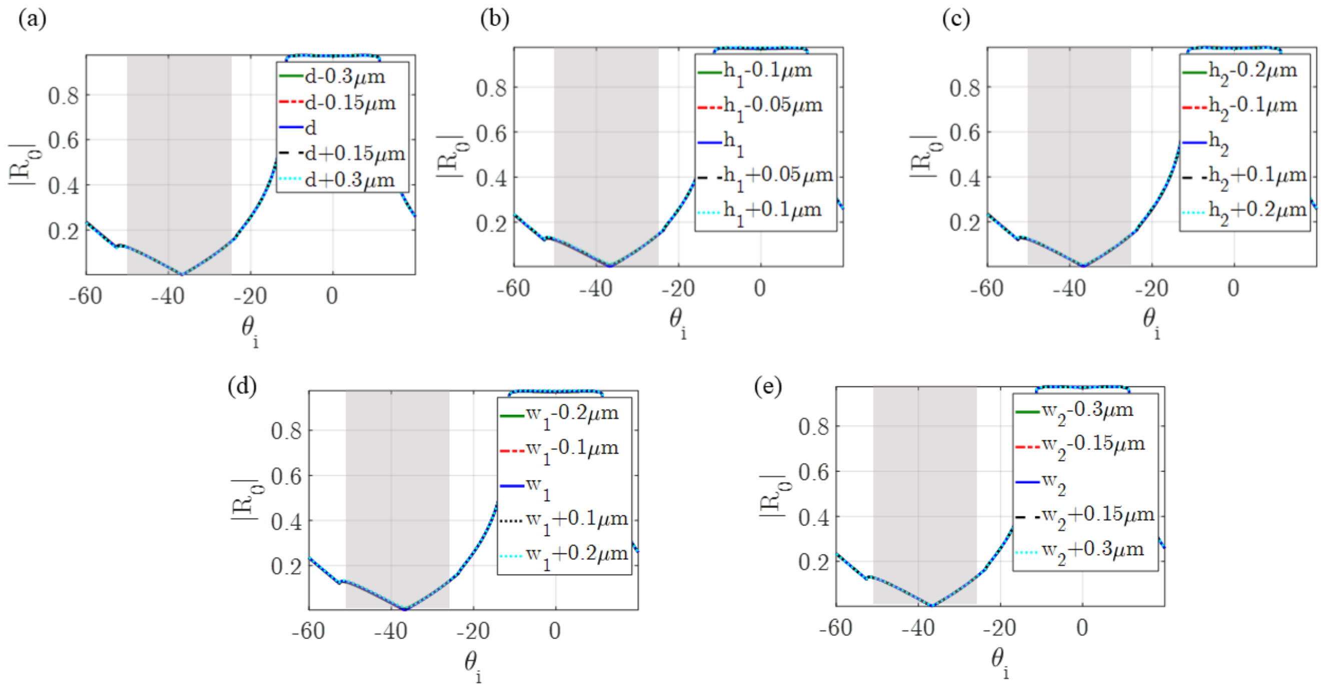

Supplementary Figure S3. Tolerance analysis. (a)-(e) The angular spectra of the amplitude, $|R_0|$, for small variations in the geometrical parameters d, h1, h2, w1, w2, respectively.

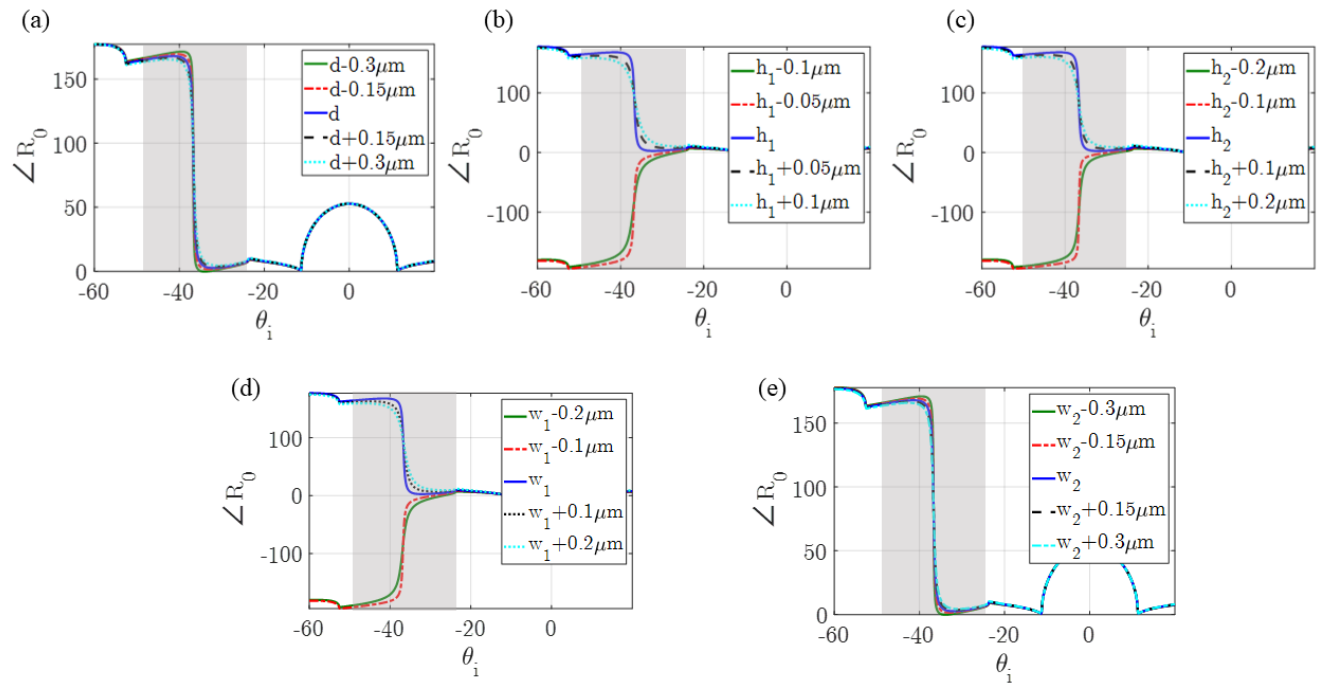

Supplementary Figure S4. Tolerance analysis. (a)-(e) The angular spectra of the phase, Arg$\{R_0\}$, for small variations in the geometrical parameters d, h1, h2, w1, w2, respectively.

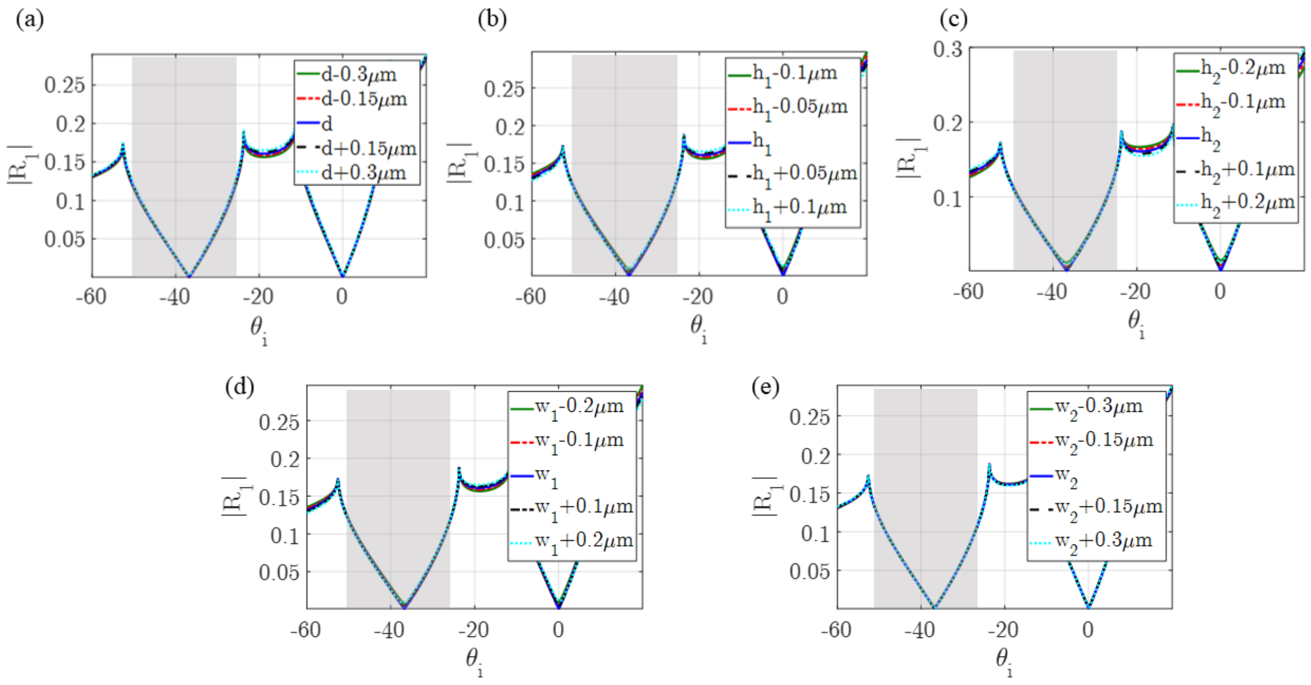

Supplementary Figure S5. Tolerance analysis. (a)-(e) The angular spectra of the amplitude, $|R_1|$, for small variations in the geometrical parameters d, h1, h2, w1, w2, respectively.

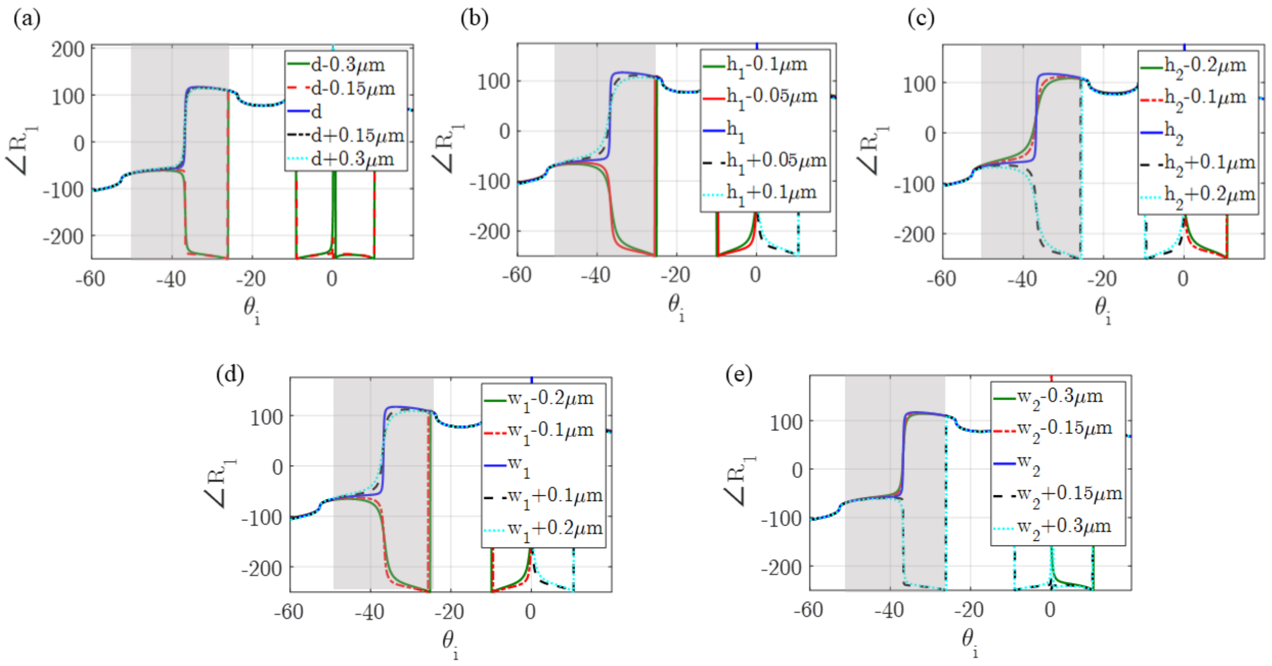

Supplementary Figure S6. Tolerance analysis. (a)-(e) The angular spectra of the phase, Arg{R_1}, for small variations in the geometrical parameters d, h1, h2, w1, w2, respectively.

Supplementary Appendix E (Image Denoising)

Image denoising is one of the most fundamental challenges in image processing and computer vision, with the purpose of estimating the original image by suppressing noise from a noise-contaminated version of the image. Image noise can be caused by a variety of intrinsic (sensor) and extrinsic (environment) factors that are difficult to prevent in the real life situations. Beside several applications of image denoising, it can be considered as a preprocessing for edge-detection, image segmentation, and image classification where recovering the specific features of original image is critical. Herein, by re-optimizing the specular mode of the metagrating shown in Fig. 4 of the paper, we present a simple image denoising demonstration through applying a sharp low-pass filter on the input image.

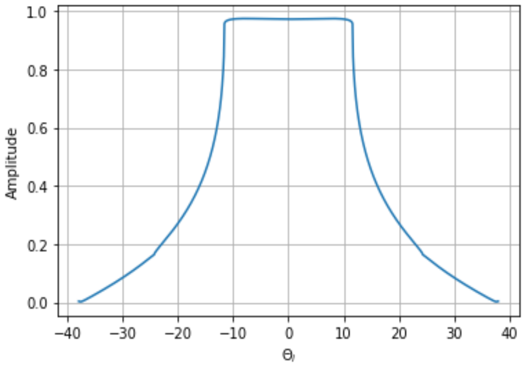

Supplementary Figure S7. Image denoising filter. The amplitude of the specular mode for the re-optimized metagrating of Fig. 4 as the transfer function of image denoising. The optimum structural parameters are $\varepsilon_{r1} = \varepsilon_{r2} = \varepsilon_{r0} = 1$, w_1 =0.026L_x, w_2=0.0476L_x, h_1=0.43L_x, h_2=0.427L_x, and d=0.538L_x.

In order to illustrate the performance of the image-denoising task, we select a grayscale image and compare the performance with the original image.

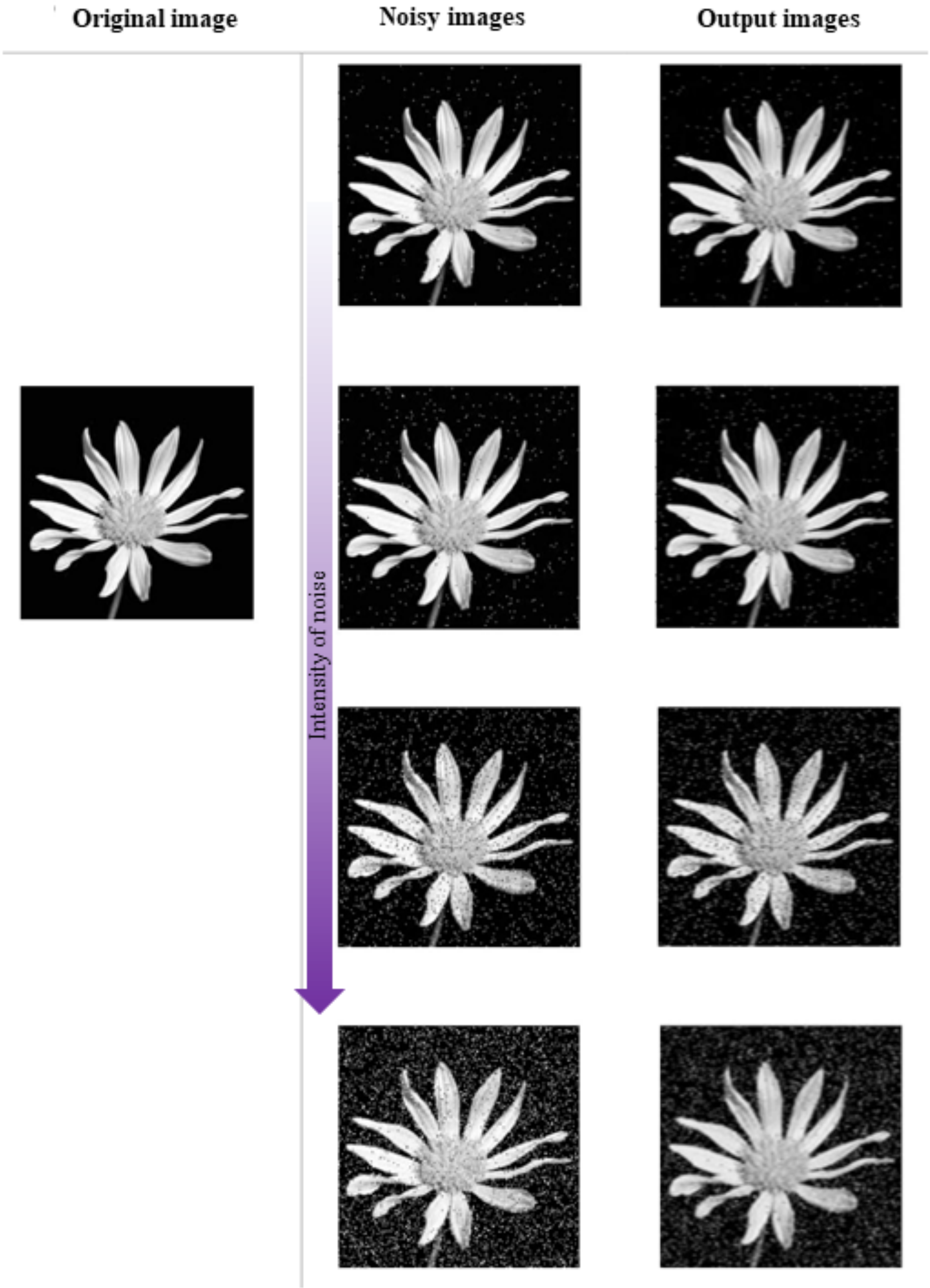

Supplementary Figure S8. The illustration of image denoising on an input image for different noise levels using the designed metagrating of Supplementary Figure S7.
